# Nationwide surveillance of AIDS-defining illnesses among HIV patients in Japan from 1995 to 2017

**DOI:** 10.1371/journal.pone.0256452

**Published:** 2021-08-19

**Authors:** Takeshi Tanaka, Kazuhiro Oshima, Kei Kawano, Masato Tashiro, Akitaka Tanaka, Ayumi Fujita, Misuzu Tsukamoto, Akira Yasuoka, Katsuji Teruya, Koichi Izumikawa

**Affiliations:** 1 Infection Control and Education Center, Nagasaki University Hospital, Nagasaki-shi, Nagasaki, Japan; 2 Department of Internal Medicine, Nagasaki Goto Chuoh Hospital, Goto-shi, Nagasaki, Japan; 3 Department of Infectious Diseases, Nagasaki University Graduate School of Biomedical Science, Nagasaki-shi, Nagasaki, Japan; 4 Department of Internal Medicine, Hokusho Central Hospital, Sasebo-shi, Nagasaki, Japan; 5 Division of Internal Medicine, Omura Municipal Hospital, Omura-shi, Nagasaki, Japan; 6 Department of AIDS Clinical Center, Center Hospital of the National Center for Global Health and Medicine, Shinjuku-ku, Tokyo, Japan; Faculty of Medicine, Chiang Mai University, THAILAND

## Abstract

**Objectives:**

The accurate prevalence of acquired immunodeficiency syndrome (AIDS)-defining illnesses (ADIs) in human immunodeficiency virus (HIV)-infected patients has not been well investigated. Hence, a longitudinal nationwide surveillance study analyzing the current status and national trend of opportunistic complications in HIV-infected patients in Japan is warranted.

**Methods:**

A nationwide surveillance of opportunistic complications in HIV-infected patients from 1995 to 2017 in Japan was conducted. An annual questionnaire was sent to 383 HIV/AIDS referral hospitals across Japan to collect information (CD4+ lymphocyte count, time of onset, outcome, and antiretroviral therapy [ART] status) of patients diagnosed with any of 23 ADIs between 1995 and 2017.

**Results:**

The response and case capture rates of the questionnaires in 2017 were 53% and 76%, respectively. The number of reported cases of opportunistic complications peaked in 2011 and subsequently declined. *Pneumocystis* pneumonia (38.7%), cytomegalovirus infection (13.6%), and candidiasis (12.8%) were associated with the cumulative incidence of ADIs between 1995 and 2017. The mortality rate in HIV-infected patients with opportunistic complications substantially decreased to 3.6% in 2017. The mortality rate was significantly higher in HIV patients who received ART within 14 days of diagnosis of complications than in those who received ART 15 days after diagnosis (13.0% vs. 3.2%, p < 0.01).

**Conclusions:**

We have demonstrated a 23-year trend of a newly diagnosed AIDS status in Japan with high accuracy. The current data reveal the importance of *Pneumocystis* pneumonia as a first-onset illness and that early initiation of ART results in poor outcomes in HIV patients in Japan.

## Introduction

As of 2018, a total of 37.9 million people were living with human immunodeficiency virus (HIV), and 770,000 people have died from HIV-related causes worldwide annually [1]. According to the Millennium Development Goals report [2], HIV infections have declined by 40% from 2000 to 2013 worldwide. In Japan, the number of newly reported cases of HIV infection or AIDS has peaked. The Ministry of Health, Labour and Welfare (MHLW) of Japan reported that approximately 30% of HIV-infected patients in Japan are unaware of their HIV infection and present with one of 23 diseases or acquired immunodeficiency syndrome (AIDS)-defining illnesses (ADIs) as the initial indicator of AIDS. These ADIs have been defined by the Japanese government for the purpose of national surveillance and reporting (S1 Table); hence, the list of diseases may partially vary from those in other countries [3–5]. This public reporting system provides information on only the first diagnosis. Information regarding the clinical course and outcomes following the initial diagnosis is not available. Therefore, there are no available studies of a nationwide surveillance of AIDS morbidity and mortality trends in Japan.

Significant improvements in the prognosis of HIV infection have been accomplished with the universal use of antiretroviral therapy (ART) [6, 7]. The expansion of ART coverage has decreased not only the incidence of AIDS and AIDS-related morbidity and mortality but also the transmission of HIV [8–12]. The mortality rate in patients who develop AIDS is high, especially in patients who do not receive ART [13]; thus, early detection of HIV/AIDS and initiation of continuous ART is a crucial factor for improving the prognosis of HIV infection [14, 15].

According to the guidelines for the use of antiretroviral agents in HIV-1-infected adults and adolescents, ART should be initiated as soon as possible in patients with ADIs. Currently, there is no effective therapy for ADIs (such as cryptosporidiosis and progressive multifocal leukoencephalopathy) because the recovery of immune function with ART may improve disease outcomes [16]. However, because immediate ART may increase the risk of severe immune reconstitution inflammatory syndrome (IRIS) in patients with some ADIs such as cryptococcal and tuberculous meningitis, a short delay before initiating ART may be warranted [16]. However, in a previous study, compared with deferred initiation, early initiation of ART resulted in improved survival rates in cases of other ADIs such as *Pneumocystis* pneumonia (PCP) [17]. In this trial, ART initiation within 14 days of initiation ADI treatment was considered early initiation and that between 42 and 84 days of initiating ADI treatment was considered deferred initiation. No other evidence recommending the appropriate time for early initiation of ART is presently available; therefore, the appropriate timing of initiating ART for patients with ADIs is unclear.

We conducted a nationwide survey funded by the Japan Agency for Medical Research and Development on the onset of AIDS, outcomes of each ADI, and time of initiating ART in patients with opportunistic ADI complications between 1995 and 2017 in Japan. This study aimed to investigate the prevalence and long-term trends of ADIs in HIV patients in Japan.

## Methods

### Subjects of this survey

In Japan, most patients diagnosed with HIV infection are referred to HIV/AIDS referral hospitals. Therefore, we targeted these hospitals for conducting the survey. These hospitals were located at 383 sites throughout Japan as of September 2017. Since 1995, we have posted an annual questionnaire on ADIs for patients diagnosed with ADIs from January to December of the previous year. The ADIs vary among countries. In this survey, we used the ADIs defined by the MHLW of Japan (S1 Table).

### Questionnaire development

The questionnaire was divided into two segments. The first part included questions on whether HIV patients with ADIs were present in each hospital. The second part included questions on detailed information regarding these patients with ADIs, which included questions on the description of ADIs, onset time of ADIs, CD4+ lymphocyte count at onset, diagnostic procedures used for ADIs, outcomes, ART initiation at onset, elapsed time from the initial HIV diagnosis until the onset of ADIs, and ART initiation time after the diagnosis of ADIs. A detailed survey of each disease was omitted because this study aimed to determine the trends of ADIs in HIV patients in Japan. The outcomes, including survival status, were recorded at the time of response to the questionnaire by each physician. Because this surveillance is an annual report, the outcome was a clinical judgment made by each physician up to a year after diagnosis. Questionnaire responses were recorded in a database created using Microsoft Access 2010 for further analysis. The questionnaire was developed in accordance with the ethical guidelines for medical research targeting human subjects [18]; therefore, it did not include identifiers such as the initials or record numbers of patients to which they could be linked. Clinical data regarding HIV infection obtained through questionnaires were carefully managed in a laboratory environment to which only the researchers in charge had access.

### Mortality rate

The mortality rate was calculated as the number of deaths reported by the survey per number of AIDS patients diagnosed in a calendar year.

### Case capture rate

The annual capture rate was calculated based on the number of AIDS cases reported to the AIDS Surveillance Committee of the MHLW of Japan. The capture rate (average of the last 2 years) was calculated as the number of AIDS cases reported through our questionnaires (total number in the last 2 years) divided by the number of AIDS cases reported through MHLW surveillance (total number in the last 2 years).

### Statistical analysis

All data were analyzed using IBM SPSS 23. The relationship between the ART initiation time after the diagnosis of ADIs and outcomes was analyzed using the Pearson chi-square test and Fisher’s exact test. Statistical significance was set at p < 0.05.

### Ethics

This study was approved by the Institutional Review Board of the Nagasaki University Hospital (approval number: 18111929–2) and by the institutional review boards of each participating hospital as required.

## Results

### Trends in the incidence of opportunistic complications

**Fig 1:**
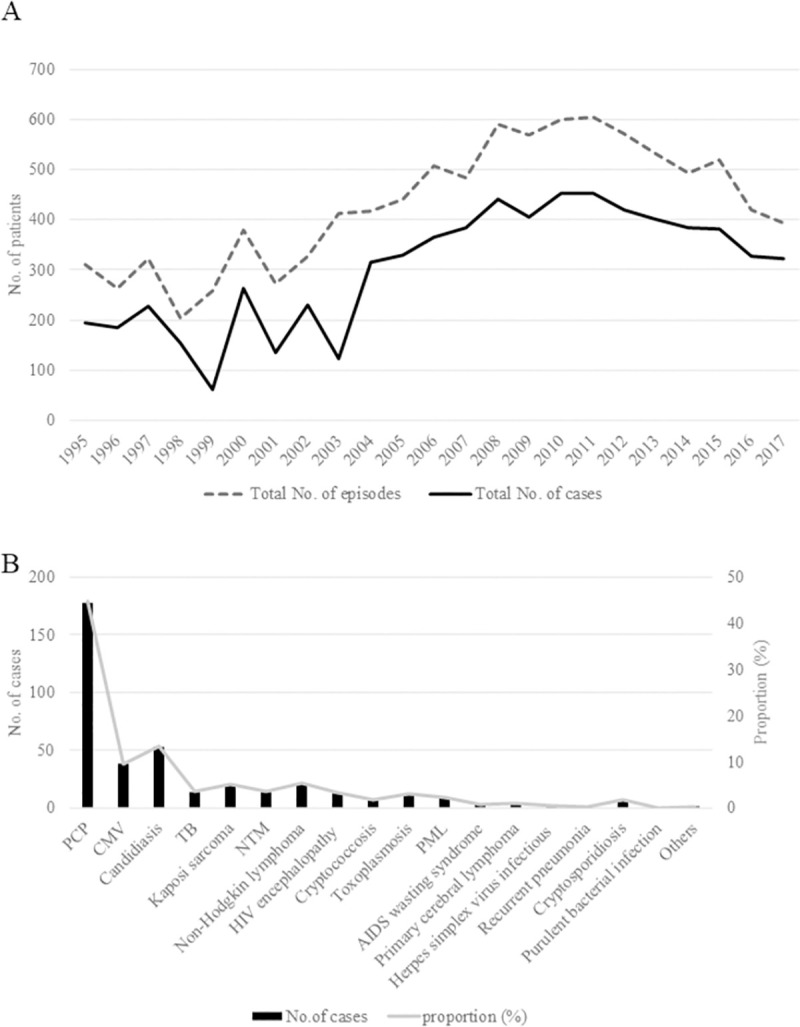
Incidence trends of acquired immunodeficiency syndrome (AIDS)-defining illnesses (ADIs). (A) Change in the number of reported cases of ADIs over time (based on the questionnaire sent to human immunodeficiency virus [HIV]/AIDS referral hospitals across Japan). The total number of patients and episodes encountered in 2017 was 320 and 391, respectively. The numbers indicate a continuously decreasing trend since 2012. (B) Cumulative incidence and proportion of ADIs between 1995 and 2017. *Pneumocystis* pneumonia (PCP) occurred the most frequently, followed by cytomegalovirus (CMV) infection, candidiasis, tuberculosis (TB), Kaposi sarcoma, and non-tuberculous mycobacterial (NTM) infection.

We posted questionnaires to HIV/AIDS referral hospitals throughout Japan annually between 1995 and 2017. The annual response rate was 55%–67%. In 2017, questionnaires were sent to 383 hospitals, and the response rate was 53%. The total number of patients and episodes encountered in 2017 was 320 and 391, respectively, reflecting a continuously decreasing trend since 2012. According to the MHLW AIDS Surveillance Committee, the number of HIV and AIDS cases was 976 and 413, respectively, in 2017, and the number of HIV and AIDS cases has been >900 and >400, respectively, since 2006. The case capture rate in the present study was estimated to be 76% in 2017. The annual trend of the case capture rate is presented in S1 Fig. The initial increase and subsequent decrease in the trend of the case capture rate was caused by variations in the annual number of questionnaire response facilities. The total number of cases with ADIs gradually increased from 178 in 1996 to 453 in 2010 and thereafter plateaued before gradually decreasing (Fig 1A). The cumulative frequencies of the ADIs between 1995 and 2017 are presented in Fig 1B. In this study, ADIs were diagnosed in 7215 HIV-infected patients. PCP occurred the most frequently, followed by cytomegalovirus (CMV) infection, candidiasis, tuberculosis (TB), Kaposi sarcoma, and non-tuberculous mycobacterial infection.

### Mortality rate of ADIs in Japan

**Fig 2:**
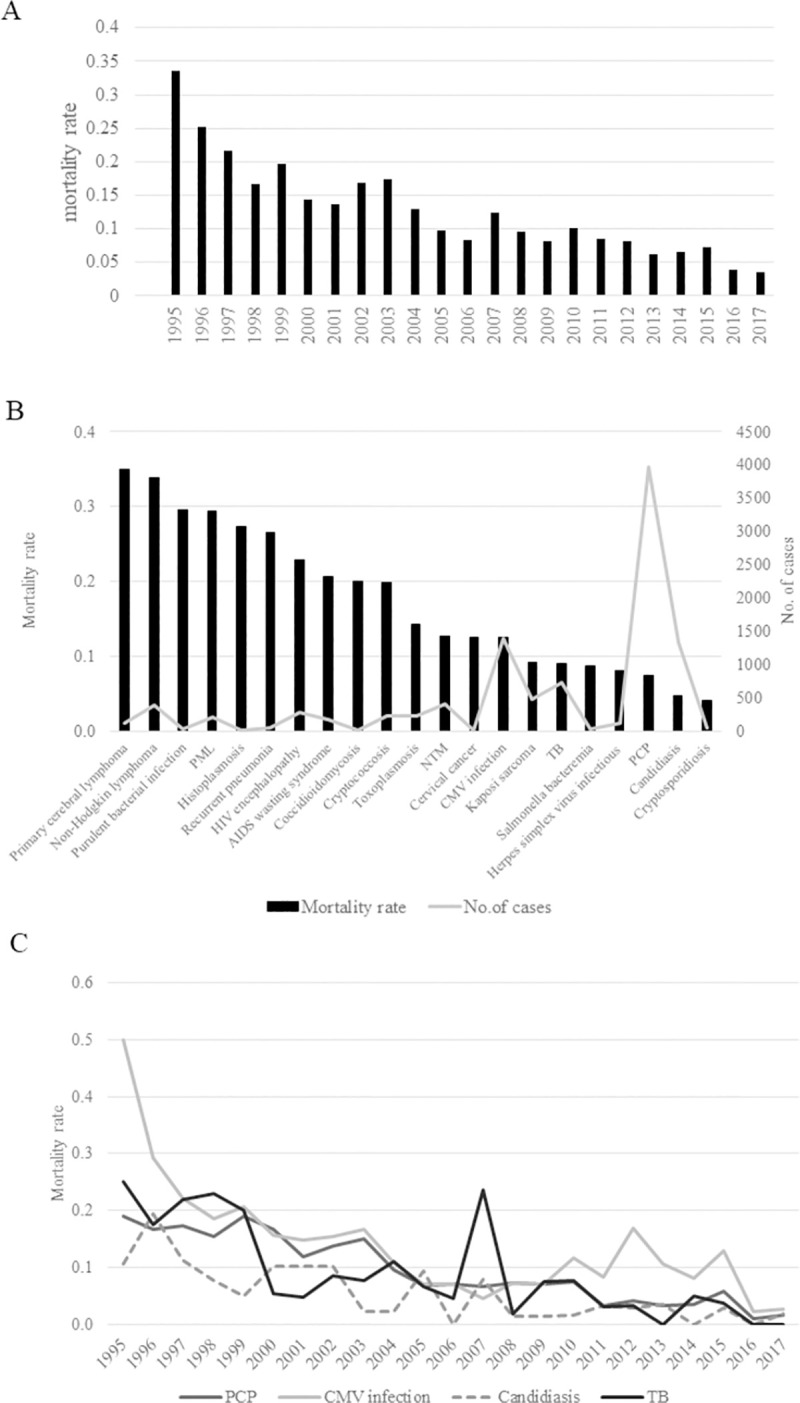
Mortality rate of acquired immunodeficiency syndrome (AIDS)-defining illness (ADIs) in Japan. (A) Annual trends of ADI mortality rates. The rate substantially decreased to 0.036 in 2017. (B) Disease-specific cumulative mortality rates and the number of patients who died. The rates of malignancies (non-Hodgkin’s lymphoma and primary brain lymphoma) and central nervous system (CNS)-related diseases such as progressive multifocal leukoencephalopathy (PML), human immunodeficiency virus (HIV) encephalopathy, and cryptococcosis were characteristically high. Among infectious diseases, histoplasmosis, purulent bacterial infections, recurrent pneumonia, and cryptococcosis were associated with high mortality. (C) Trend of the mortality rates of four major diseases—*Pneumocystis* pneumonia (PCP), cytomegalovirus (CMV) infection, candidiasis, and tuberculosis. The mortality rates of all four diseases decreased over time. The survival status was recorded at the time of response to the questionnaire as a clinical judgement made by each physician up to a year after diagnosis.

The mortality rates of patients who developed ADIs are presented in Fig 2. The mortality rate in patients who developed ADIs was >0.25 in 1995. However, the mortality rate gradually decreased and fluctuated between 0.06 and 0.1 from 2005, except in 2007, reaching 0.038 in 2016 and 0.036 in 2017 (Fig 2A).

Regarding the cumulative mortality rates of AIDS (Fig 2B), the mortality rates of malignant tumors (non-Hodgkin lymphoma and primary cerebral lymphoma) and central nervous system diseases such as progressive multifocal leukoencephalopathy (PML), HIV encephalopathy, and cryptococcosis were higher than those of other diseases. Furthermore, a high mortality rate was observed for infectious diseases, such as histoplasmosis, purulent bacterial infection, recurrent pneumonia, and cryptococcosis.

We analyzed the change in mortality rates of four major diseases—PCP, CMV infection, candidiasis, and TB, by year and found that the rates of all four diseases decreased over time (Fig 2C). In recent years, the mortality rate of PCP and TB has been similar to that of candidiasis, which has been at its lowest level. The mortality rate of CMV infection continuously decreased from 1995 to 2005 and has remained unchanged since 2005.

### Status of HIV diagnosis and ART at the onset of ADIs

**Fig 3:**
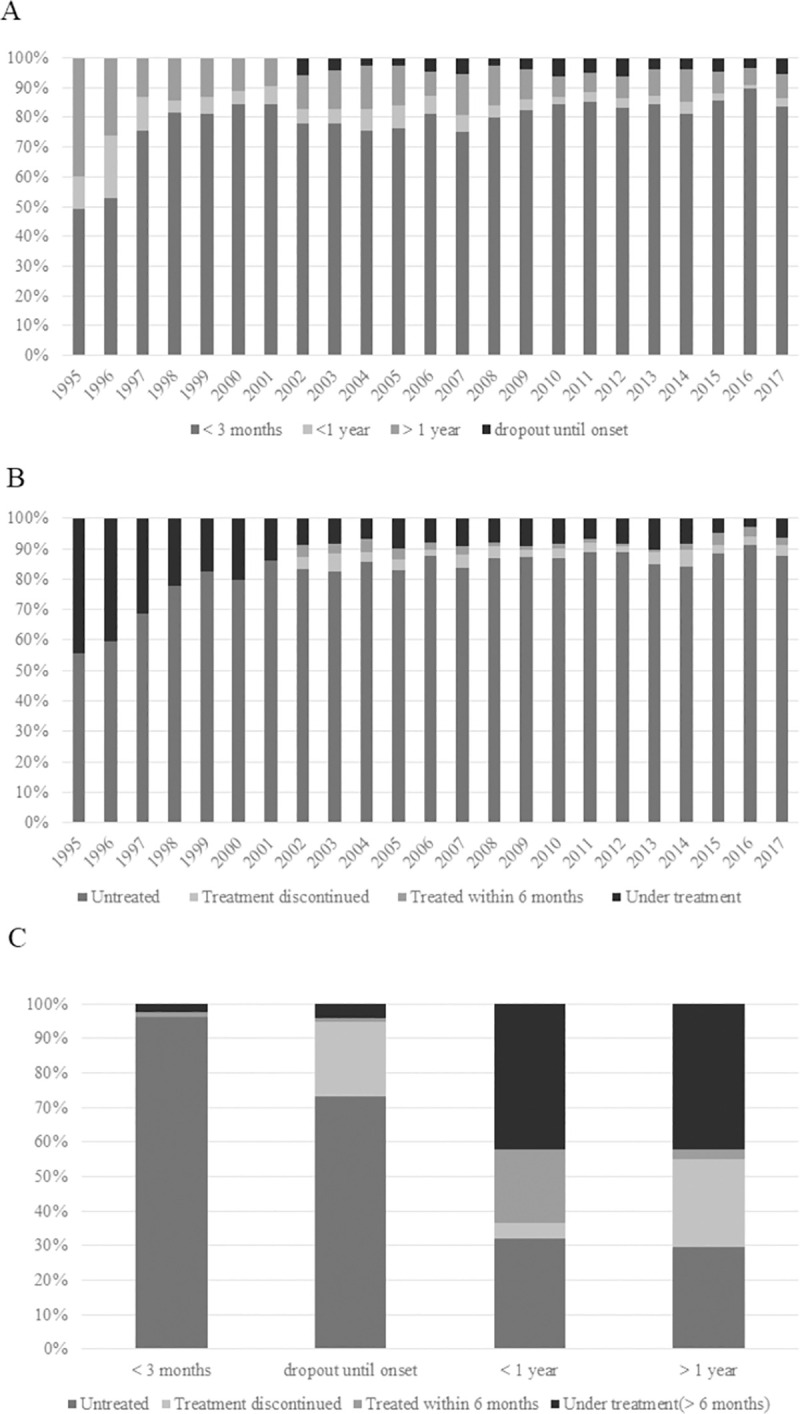
Status of human immunodeficiency virus (HIV) diagnosis and antiretroviral therapy (ART) at the onset of AIDS-defining illnesses (ADIs). (A) Time from HIV diagnosis to the onset of ADIs. The largest proportion of patients with ADIs were diagnosed within 3 months before and after HIV diagnosis. (B) Status of ART at the onset of ADIs. Most (87.8%) patients were not receiving ART at the onset of ADIs. (C) Time from HIV diagnosis to symptom onset (2002–2017). Relationship between time from HIV diagnosis to the onset of ADIs and use of ART. Most patients diagnosed with ADIs within 3 months after HIV diagnosis and those dropped out until ADI onset were untreated or had discontinued treatment.

Analysis of the time from the initial HIV diagnosis to the onset of ADIs revealed that patients who developed ADIs within 3 months after HIV diagnosis and those who were diagnosed with ADIs before HIV diagnosis accounted for the majority of patients with ADI onset in 1997 and thereafter, which corresponds to the time when ART became universally available (Fig 3A). The number of patients who developed ADIs within 3 months after HIV diagnosis constituted the highest proportion until 2017. Among these, some patients were considered to have been diagnosed with HIV and AIDS simultaneously. The percentage of patients unexamined for a long period (labeled “dropout until onset” in Fig 3A) remained unchanged since 2002 (Fig 3A). The data of patients who were unexamined for a long period (“dropout until onset”) have been included since 2002.

As of 2017, most (87.8%) patients had not received ART at the onset of ADIs since 1999 (Fig 3B).

Cross-tabulation analysis of the time from HIV diagnosis to the onset of ADIs and the duration of anti-HIV treatment was performed using cumulative data since 2002 (Fig 3C). The results indicated that among patients who developed ADIs within 3 months after HIV diagnosis and those who were unexamined for a long period, most patients were untreated or had discontinued the treatment. In fact, among patients who experienced an onset of ADIs after a year or more of HIV diagnosis, nearly 60% did not receive treatment or discontinued treatment. Among these patients, 24.6% discontinued treatment and 41.7% continuously received ART for at least 6 months.

### Time trends in ART initiation

**Fig 4:**
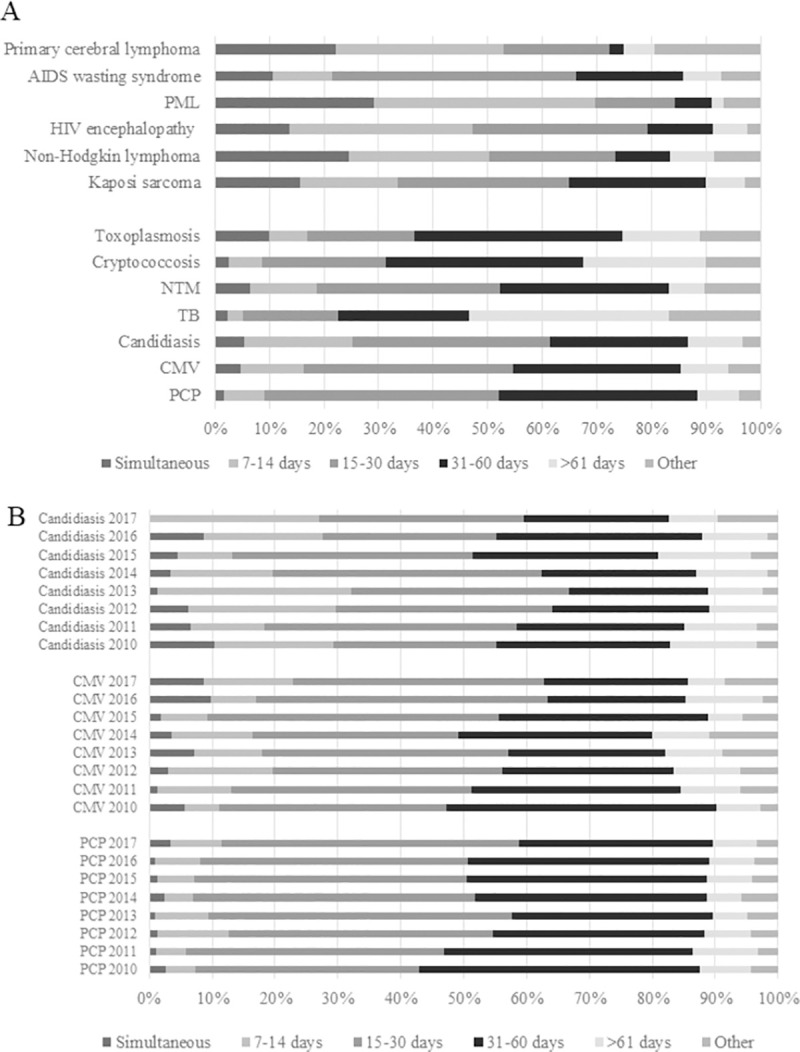
Time trends of antiretroviral therapy (ART) initiation. (A) Time to ART initiation after diagnosis of acquired immunodeficiency syndrome-defining illnesses (ADIs): cumulative data from 2010 to 2017. Regarding infectious diseases, ART was mostly introduced 15–30 days after malignancies and non-infectious encephalopathy were detected. Particularly for tuberculosis (TB), more than half of the patients initiated ART >2 months after HIV diagnosis. ART was mostly initiated earlier for central nervous system (CNS)-related diseases such as progressive multifocal leukoencephalopathy (PML) and primary cerebral lymphoma with approximately 60% and 80%–90% human immunodeficiency virus (HIV) patients initiated ART within 14 and 30 days after diagnosis, respectively. (B) Time to ART initiation after the diagnosis of ADIs (three major infectious diseases): an 8-year comparison (2010–2017). Most HIV patients received ART >15 or >31 days after diagnosis.

Fig 4A shows the time of ART initiation after the diagnosis of ADIs. ART was introduced >15 days after the diagnosis of ADIs such as infectious diseases, unlike ADIs such as malignant tumors and noninfectious encephalopathy. ART was initiated >2 months after diagnosis in nearly 50% patients diagnosed with active TB. Although the number of patients with central nervous system diseases such as PML and primary cerebral lymphoma was small, ART was introduced within 14 days in approximately 60% patients and within 30 days in 80%–90% patients, indicating a trend of early initiation.

A trend of early initiation of ART was observed from 2010 to 2017 in patients with three major infectious diseases (candidiasis, CMV infection, and PCP). Most HIV patients received ART >15 or >31 days after diagnosis. The percentage of patients who received ART within 14 days after diagnosis did not change significantly (Fig 4B).

### Effects of early ART initiation in patients with ADIs

The relationship between ART initiation time and outcomes for different ADIs is presented in Table 1. The mortality of all ADIs was significantly higher among patients who received ART within 14 days (13.0%) than among those who received it >15 days after diagnosis of ADIs (3.2%) (p < 0.01). Although patients were grouped based on a 30-day period of diagnosis, the death percentage was significantly higher in the group that received ART within 30 days (6.5%) than in the group that received ART after 31 days of ADI diagnosis (3%) (p < 0.01). The mortality rate of opportunistic infections (OIs), excluding ADIs such as malignant diseases, was significantly higher in the group that received ART within 14 days (9.2%) than in the group that received it >15 days after diagnosis of ADIs (2.4%) (p < 0.01). Although patients were grouped based on a 30-day period of diagnosis, the death rate was significantly higher in the group that received ART within 30 days (4.4%) than in the group that received ART after >31 days of diagnosis of ADIs (2.2%) (p < 0.01). The analysis by disease revealed that in PCP and CMV infection, the mortality rate was significantly higher in the group that received ART within 14 days (8.2% and 14.0%, respectively) than in the group that received ART >15 days after diagnosis of ADIs (1.7% and 5.8%, respectively) (p < 0.01 and p < 0.01, respectively). However, no significant difference was observed in the mortality rate when patients were grouped based on a 30-day period of ART initiation after diagnosis.

**Table 1:** Relationship between time to ART introduction and outcomes (2010–2017).

		0–14 days	>15 days	p value	0–30 days	>31 days	p value
Total	Death	84 (13.0%)	92 (3.2%)	<0.01	131 (6.5%)	45 (3.0%)	<0.01
	Other than death	563 (87.1%)	2765 (96.8%)	NS	1875 (93.5%)	1453 (97.0%)	NS
Overall (only infection)	Death	41 (9.2%)	63 (2.4%)	<0.01	73 (4.4%)	31 (2.2%)	<0.01
	Other than death	405 (90.8%)	2521 (97.6%)	NS	1579 (95.6%)	1347 (97.6%)	NS
PCP	Death	12 (8.2%)	25 (1.7%)	<0.01	24 (2.7%)	13 (1.7%)	NS
	Other than death	135 (91.8%)	1479 (98.3%)	NS	862 (97.3%)	752 (98.3%)	NS
CMV	Death	9 (14.0%)	19 (5.84%)	<0.01	19 (8.1%)	9 (6.0%)	NS
	Other than death	44 (85.9%)	274 (94.2%)	NS	174 (91.9%)	144 (94.0%)	NS

Abbreviations: ART, antiretroviral therapy; CMV, cytomegalovirus; PCP, *Pneumocystis* pneumonia; NS, not significant

## Discussion

The results of surveys conducted from 1995 to 2017 suggest that the incidence of AIDS has been decreasing since 2012. Based on the publicly reported numbers of ADI cases by the AIDS Surveillance Committee Japan, we calculated the capture rate of many ADIs in our survey (approximately 80%), which allowed us to interpret the trends of OIs complications. The incidence of ADIs has been decreasing over the last few years, but it is necessary to continuously monitor the trends for fluctuations. ADIs mainly occur in patients who have not been diagnosed with HIV or have not received ART.

The prognosis of HIV infection has significantly improved owing to the advancement and widespread implementation of ART. Furthermore, the mortality rates of the four major diseases—PCP, CMV infection, candidiasis, and active TB—have significantly improved because of the enhanced recognition of these diseases by physicians and advanced diagnostic techniques. In particular, the mortality rates of three of these diseases (PCP, candidiasis, and active TB), excluding CMV infection, have been low (3%–4%) in recent years.

The overall mortality rate in patients at AIDS onset was between 0.08 and 0.09, and the high mortality rate did not change until 2012. The mortality rate declined to 0.06–0.07 for two consecutive years from 2013 and finally dropped to nearly 0.03 in 2016 and 2017. Since HIV infection has become a chronic disease, it is important from an epidemiological point of view to monitor the disease and determine an index that ensures that the mortality rate of OIs will also reach zero on account of advances in therapeutic agents and disease management. Evidence that serves as an indicator is essential in making such decisions. Therefore, continuous tracking of the trend is necessary. In ADIs such as malignant tumors, particularly non-Hodgkin`s lymphoma or Kaposi sarcoma, the number of cases shows an increasing trend. In addition, the mortality rates of central nervous system diseases such as primary cerebral lymphoma and PML were high, although the diseases were not common.

Because the pathogenesis of HIV infection involves the breakdown of immune tolerance, especially with regard to CD4+ lymphocytes, lowering the number of CD4+ lymphocytes (200–500 cells/mL). is a critical factor for initiating ART [19–21]. However, several recent large trials have addressed the benefit of early, rather than deferred, initiation of ART [14, 22]. Because the aforementioned studies are fundamental to the recommendation for early initiation of ART, whether HIV is diagnosed early in the disease clinical course before it progresses to AIDS is usually a critical issue.

Most current guidelines and many clinicians opine that ART should be initiated as soon as possible after the onset of ADIs [16]. However, ART should be initiated with caution in specific populations based on the type of infection.

No clear conclusion has been reached regarding the optimal time for ART initiation for patients with ADIs. Deferred ART initiation is a reasonable approach to prevent IRIS; however, it might increase the risk of OIs. According to the guidelines for the prevention and treatment of OIs in adults and adolescents with HIV [16], for ADIs for which no effective therapy has been established, such as PML and cryptosporidiosis, ART should be initiated without delay; however, for ADIs with concerns regarding severe IRIS, such as cryptococcal and TB meningitis, short delayed or deferred, rather than early, ART initiation is recommended because of the high risk of mortality [23–26]. Zolopa et al. reported that in the management of AIDS, compared to initiation of ART after completion of acute OI treatment (within 42–82 days), early initiation of ART (within 14 days of starting OI treatment) in patients presenting with ADIs (PCP, 63%; cryptococcal meningitis, 12%; bacterial infection, 12%) resulted in better outcomes [17]. Based on the study by Zolopa et al., we compared the prognosis of OIs between the group that received ART within 14 days and the group that received it after 15 days of diagnosis of ADIs and between the group that received ART within 30 days and the group that received it after 31 days of diagnosis of ADIs. Contrary to the findings of previous studies, our study revealed that the analysis of the relationship between the time of ART initiation and outcomes revealed that early initiation of ART did not necessarily improve prognosis.

Owing to the retrospective design of the questionnaire, this study has some limitations. First, a response rate of 53% from all HIV/AIDS referral hospitals may have led to a selection bias. However, most of the high-volume centers in each district of Japan responded to our survey every year and the case capture rate was relatively high (around 80%); therefore, we do not believe there was a large selection bias. Second, the questionnaire items were minimized in content for simplicity and ease of completion; therefore, little background information of patients was obtained to perform additional comparative analysis. Second, our prognosis analysis was performed without adjustment for age, sex, CD4 count, and other markers of health status. Therefore, it may be reasoned that the higher reported death rate of patients starting ART earlier is a result of these patients being more unwell at the time of AIDS diagnosis. Third, prognosis analysis for ADIs other than PCP and CMV infection could not be performed because of inadequate data to conduct power analysis. The data in Table 1 might be influenced by the aforementioned limiting factors. Furthermore, since this survey employs an annual questionnaire-based method, follow-up information for each case could not be obtained; therefore, the survival curve could not be generated. Some studies whose findings that partially support our results are as follows. Deconinck et al. analyzed 437 patients (PCP [37%], TB [24%], toxoplasmosis [12%], and Kaposi sarcoma [11%]) and demonstrated that there was no difference in disease progression between patients who received deferred ART (by ≥30 days) and those who received early ART. Patients in the deferred group had fewer ART modifications and shorter inpatient stays than patients in the early ART group [27]. Schäfer et al. compared early ART (within 7 days after initiation of ADI treatment) and deferred ART (ART after completion of ADI treatment) in 61 patients (toxoplasma encephalitis, 11 patients; PCP, 50 patients) and reported that there were no differences in the incidence of disease progression or IRIS [28]. Although there is no established consensus regarding the appropriate time of ART initiation for patients with ADIs, it is reasonable to consider the association between IRIS and early ART initiation. The prognosis of severe PCP is improved when steroids are employed at treatment initiation [29]. This suggests that the prognosis of PCP is related to an enhanced immune response after treatment. This may mean that early ART induction may worsen prognosis by enhancing the immune response, especially in PCP. As mentioned above, in cryptococcal meningoencephalitis, there is a consensus that early ART initiation worsens prognosis and that the appropriate timing of ART initiation should be determined on a disease-specific basis. Our results indicate that early ART initiation may be associated with poor prognosis, which needs to be validated in future well-designed clinical trials. Our findings are one of the few findings demonstrating the poor outcomes of early ART in ADIs. Although we have shown the outcome analysis of PCP and CMV infection, continuous collection of national data is worthwhile for establishing evidence regarding the optimal time for ART initiation in the remaining ADIs. In conclusion, our data, based on high-volume information obtained via a national surveillance program, indicated the most accurate 22-year trends of HIV/AIDS prognosis and ART status in Japan. We have identified a unique parameter to consider—the time of ART initiation. Our survey is a valuable resource because of its sustainability in collecting serial data from Japan. Furthermore, a continuous analysis of future trends and outcomes of diseases is warranted.

## Supporting information

S1 Fig Annual trend of capture rates. Capture rate was calculated using the following formula: Capture rate (average of the last 2 years) = number of AIDS cases reported through our questionnaires (total in the last 2 years)/number of AIDS reported to MHLW surveillance (total in the last 2 years).
(TIF)

S1 Table AIDS-defining illnesses defined by the ministry of health labour and welfare of Japan. (TIF)
