# Supplementary figures and images for: Nationwide surveillance of AIDS-defining illnesses among HIV patients in Japan from 1995 to 2017
Source: PLoS One. 2021 Aug 19;16(8):e0256452. doi: 10.1371/journal.pone.0256452 (PMC8376045; doi:10.1371/journal.pone.0256452)

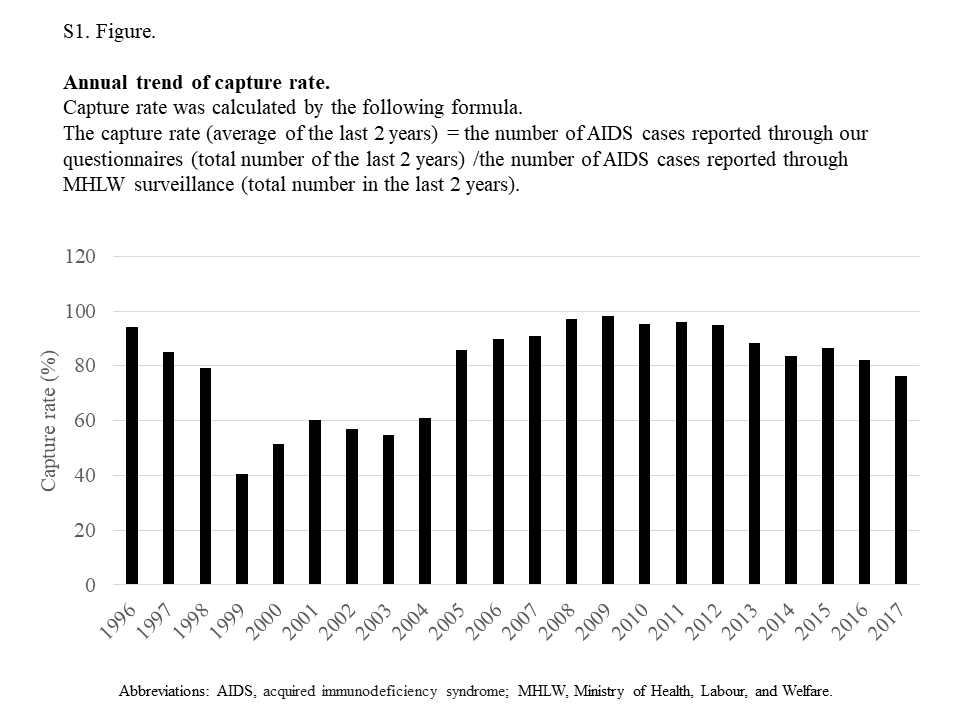

Supplement: S1 Fig — Capture rate was calculated using the following formula: Capture rate (average of the last 2 years) = number of AIDS cases reported through our questionnaires (total in the last 2 years)/number of AIDS reported to MHLW surveillance (total in the last 2 years). (TIF) [file pone.0256452.s001.tif]

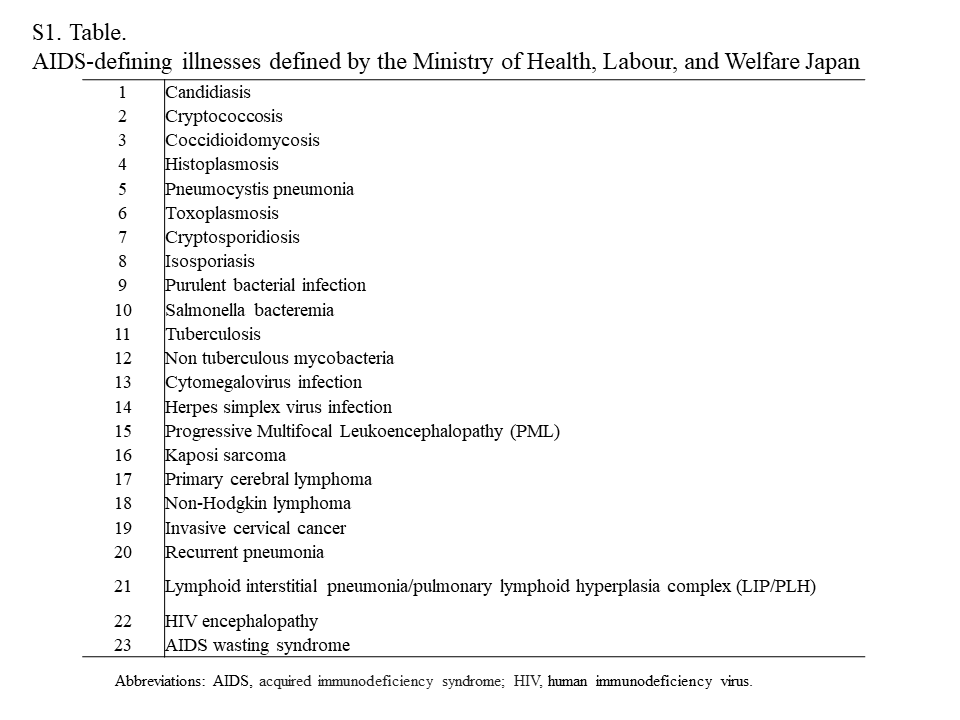

Supplement: S1 Table — (TIF) [file pone.0256452.s002.tif]
